# Editorial Comments to the Special Issue: “*Aggregaterbacter actinomycetemcomitans*—Gram-Negative Bacterial Pathogen”

**DOI:** 10.3390/pathogens9060441

**Published:** 2020-06-04

**Authors:** Jan Oscarsson, Joseph DiRienzo, Anders Johansson

**Affiliations:** 1 Department of Odontology, Umeå University, S-901 87 Umeå, Sweden; jan.oscarsson@umu.se; 2 Department of Basic and Translational Sciences, School of Dental Medicine, University of Pennsylvania, Philadelphia, PA 19104-6030, USA; dirienzo@upenn.edu

**Keywords:** *Aggregatibacter actinomycetemcomitans*, leukotoxin, cytolethal distending toxin, lipopolysaccharides, cytokine binding factors, horizontal gene transfer, outer membrane vesicles, biofilm, proteomics

## Abstract

*Aggregatibacter actinomycetemcomitans* is a periodontal pathogen colonizing the oral cavity in many individuals of the human population. It is equipped with several potent virulence factors that can cause cell death and induce or evade the host inflammatory response. Both harmless and highly virulent genotypes of the bacterium have emerged because of the large genetic diversity within the species. The oral condition and age, as well as the geographic origin of the individual, influence the risk to be colonized by a virulent genotype of the bacterium. In the present editorial, the different genetic and virulence properties of *A. actinomycetemcomitans* will be addressed in relation to the publications in this Special Issue.

## 1. Overview

In the light of three different reviews, various aspects of the pathobiont *Aggregatibacter actinomycetemcomitans* have been addressed. The role of this gram-negative facultatively anaerobic bacterium is described by Fine and co-workers [1]. They proposed that the host response can confine local damage by restricting bacteremic translocation of members of the oral microbiota to distant organs, thus constraining the morbidity and mortality of the host. The battery of virulence factors released from *A. actinomycetemcomitans*, as well as the many virulence mechanisms, support that the local and systemic host defense is a life insurance for the colonized individuals. The many weapons that the bacterium can use to evade host response and induce disease are reviewed by Belibasakis and collaborators [2]. Unique for *A. actinomycetemcomitans* among the oral bacteria is the production of two exotoxins, a leukotoxin (LtxA) and a cytolethal distending toxin (CDT). LtxA is considered to be a major virulence factor in aggressive forms of periodontitis, where it protects the bacterium from phagocytosis by killing the defense cells and induces a pro-inflammatory response in exposed macrophages. CDT can enter the nucleus and cleave double stranded DNA in the host cells, which leads to a prompt growth arrest. In an alternative mode of action, the toxin functions as a phosphatase that can contribute to the progression of an apoptotic pathway.

Both toxins can be secreted from the bacteria to the surrounding tissues and can also be spread through the release of toxin loaded outer membrane vesicles. *A. actinomycetemcomitans* is one of the human pathogens that can bind host cytokines, such as IL-1β, IL-8, and IL-6, and internalize them, which leads to changes in the biofilm properties, decreasing the metabolic activity and changing the composition of the extracellular matrix. In polymicrobial biofilm models, it has been shown that the presence of *A. actinomycetemcomitans* changes the profile of the secreted proteins. Due to the large genetic diversity of *A. actinomycetemcomitans*, there is a substantial intraspecies variation in the ability to produce virulence factors, an aspect reviewed by Nørskov-Lauritsen [3]. It is no doubt that this bacterium exists as both harmless commensals, as well as opportunistic pathogens with strong pathogenic potential. The well-studied JP2 genotype of this bacterium has enhanced production of LtxA and is shown to significantly increase the risk for onset of periodontitis in the colonized individuals. *A. actinomycetemcomitans* can be detected by both culture and PCR techniques and the characteristic 530-bp deletion in the leukotoxin promoter of the JP2 genotype function as a suitable marker for detection of highly leukotoxic genotypes. The prevalence of this bacterium is estimated to be 20% for periodontally healthy juveniles, 36% for healthy adults, 50% for adult periodontitis patients, and 90% for young periodontitis patients. The highly leukotoxic JP2 genotype, together with other virulent genotypes, are mainly detected among the young periodontitis patients.

## 2. Clinical Findings

The JP2 genotype of *A. actinomycetemcomitans* with enhanced leukotoxic activity is mostly present in individuals of North and West African origin, however, with the increasing number of reports of detection also in individuals of non-African origin [3]. In one paper of this Special Issue, two cases of Caucasians diagnosed with the JP2 genotype are presented. Both are middle-aged females with severe periodontitis [4]. Microbiological diagnostics revealed the presence of *A. actinomycetemcomitans* JP2 genotype, but not *Porphyromonas gingivalis* in the subgingival samples from the patients. Both patients were successfully treated with adjunctive antibiotics and the JP2 genotype was eliminated. The authors claim that the microbiological diagnosis was the key for the successful treatment with adjunctive antibiotics.

## 3. Phenotypic and Genotypic Characterization

The JP2 genotype of *A. actinomycetemcomitans* is the classical pathogen with enhanced leukotoxin production and enhanced prevalence in subgingival sites of adolescents with periodontitis [2]. By further genotypic and phenotypic characterization of isolates from periodontitis patients living in Sweden, another virulent type of this bacterium, *cagE*, was discovered by Johansson and collaborators [5]. This genotype is, like JP2, exclusively found in serotype b and has an enhanced leukotoxicity, but frequently lacks the JP2-like 530 bp leukotoxin promoter deletion. Interestingly, however, all hitherto identified JP2 genotype strains share the *cagE* genotype. The authors suggested therefore, and due to other common genetic features, that this genotype may be the ancestor of JP2.

The *A. actinomycetemcomitans* genome can be divided into an accessory gene pool that is found in some strains, and a core gene pool, found in all strains. Tjokro and co-workers [6] hypothesize that the accessory genes confer critical functions for the bacterium in vivo. The authors showed that these genes exhibited distinct patterns of expression from the core genes and may play a role in the survival of *A. actinomycetemcomitans* in nutrient-limited environments. These genes coded for some important virulence factors and were located both in genomic islands and in the chromosome.

Whole genome sequencing (WGS) has been introduced for characterization of the *A. actinomycetemcomitans* and has separated the species into five different clades [3]. In the largest study, 30 oral human *A. actinomycetemcomitans* strains were sequenced and characterized. In their paper within this Special Issue, Nedergard et al. [7] reported on the serotyping and genome sequencing of 29 strains of *A. actinomycetemcomitans* cultured from blood stream infections of patients in Denmark. The authors concluded that WGS data add valuable information for further classification. They suggested that the population structure of this bacterium is more accurately described by a division of the species into three phylogenetic lineages, I–III.

## 4. Host–Parasite Interactions

The host response induced by *A. actinomycetemcomitans* is a challenge for the host and mimic several mechanisms that are associated with processes of degenerative diseases, such as periodontitis [1,2]. In aggressive forms of periodontitis, the dysbiotic microbiota in the subgingival crevice is abundant in *A. actinomycetemcomitans.* Ando-Sugimoto et al. [8] report in this Special Issue that the interactions of the bacterium, with extra-and intracellular receptors of host cells, leads to exacerbated inflammation and subsequent tissue destruction. In interactions with macrophages and human gingival epithelial cells, *A. actinomycetemcomitans* induces a signaling cascade that is involved in inflammasome and inflammatory responses. Differences in host cell responses between gingival epithelial cells and macrophages led the authors to suggest that survival of *A. actinomycetemcomitans* in periodontal tissues may be favored by its ability to differentially activate host cells. The most well-studied virulence factor expressed by this bacterium is LtxA, which specifically interacts with human leukocytes [1,2]. Several important discoveries on this toxin have been made by Edward (“Ned”) Lally, who sadly passed away last year, and is a topic of an In Memoriam in this issue [9]. Lally and his co-workers discovered the receptor for LtxA (LFA-1), as well as mapped the domains of the toxin that were responsible for the interaction with its receptor. These findings explained the specificity of LtxA for leukocytes and have created a foundation for the subsequent delineation of the mechanisms in which the toxin affect various target cells. In one paper of this Special Issue, Lally et al. [10] show that LtxA enters the cytosol of lymphocytes without evidence of plasma membrane damage, utilizing receptor-mediated endocytic mechanisms. The authors suggest that LtxA can accompany LFA-1 in its recycling pathway and apparently dissociate from the receptor in endocytic vesicles and independently follow the degradative pathway. LtxA-delivery to the terminal point of this route results in the lysosomal membrane rupture. The ability of *A. actinomycetemcomitans* to express LtxA is closely linked to the capacity to kill leukocytes [2]. There is no golden standard available for quantification of LtxA production in different strains from this bacterium. Jensen and co-workers [11] applied three different methods for analyses of LtxA production in a collection of serotype b isolates. The results showed that that the JP2 genotype of *A. actinomycetemcomitans* had an enhanced LtxA production, even though there was a substantial variation between the examined isolates. The production of LtxA in the non-JP2 serotype b isolates was, in general, lower and that of JP2. However, a few non-JP2 isolates exhibited a higher LtxA production than some of the JP2 isolates. They also found a significant correlation between the levels of transcription of the leukotoxin genes and of the produced LtxA protein.

As described above, the CDT is the other exotoxin produced by *A. actinomycetemcomitans* [2]. In their paper in the present Special Issue, Shenker and co-workers [12] report on the mechanism for CDT-induced apoptosis in T-lymphocytes. Their data demonstrate that toxin-induced apoptosis is dependent upon increased levels of specific intracellular signaling, and conclude that that the ability of CDT to impair lymphocyte proliferation and promote cell death therefore compromises the host response to CDT-producing organisms. The effect of *A. actinomycetemcomitans* on epithelial cells has also been examined in a paper of this Special Issue [13]. Beklen and collaborators show that *A. actinomycetemcomitans* biofilms release outer membrane vesicles, which can be found in close contact with the epithelium. After exposure to the bacterial products, gingival epithelial cells might lose their membrane integrity and become more vulnerable to bacterial infection. In the case of an *A. actinomycetemcomitans* infection with a highly virulent variant of the bacterium, the treatment strategy often involves antibiotics [4]. Akkaoui and co-workers [14] demonstrated the efficiency of an essential oil from the plant *Origanum vulgare* in preventing *A. actinomycetemcomitans* growth in vitro. The authors found that this effect of the oil was substantially stronger than that of the tested antibiotics. The oil did not inhibit the leukotoxicity of *A. actinomycetemcomitans*, but the authors demonstrated the possibility of including leukotoxin neutralization agents in mixtures with the oil.

## 5. Conclusions

*A. actinomycetemcomitans* is a pathobiont that expresses several virulence factors with the capacity to cause imbalance in the host response and subsequently disease. The large genetic diversity of the bacterium related to its pathogenicity varies from harmless to highly virulent. *A. actinomycetemcomitans* could be isolated from the oral cavity of in many individuals of the human population, but can also sometimes be found in the bloodstream. In the oral cavity, this species is an important cause of periodontitis that affects young individuals and it is also associated with an increased risk for several systemic diseases. The present Special Issue provides insights into the various characteristics and activities *A. actinomycetemcomitans.* This information will enhance the development of valuable tools for limiting the negative health effects associated with this bacterium.
